# Rab1 GTPases as oncogenes

**DOI:** 10.18632/aging.100849

**Published:** 2015-11-18

**Authors:** Yue Li, Hui-Yun Wang, X.F. Steven Zheng

**Affiliations:** Rutgers Cancer Institute of New Jersey and Department of Pharmacology, Robert Wood Johnson Medical School, Rutgers, the State University of New Jersey, New Brunswick, NJ 08903, USA

**Keywords:** Rab1, mTOR, Hepatocellular Carcinomas (HCC), Colorectal Cancer (CRC), nutrient signaling

**Figure 1:**
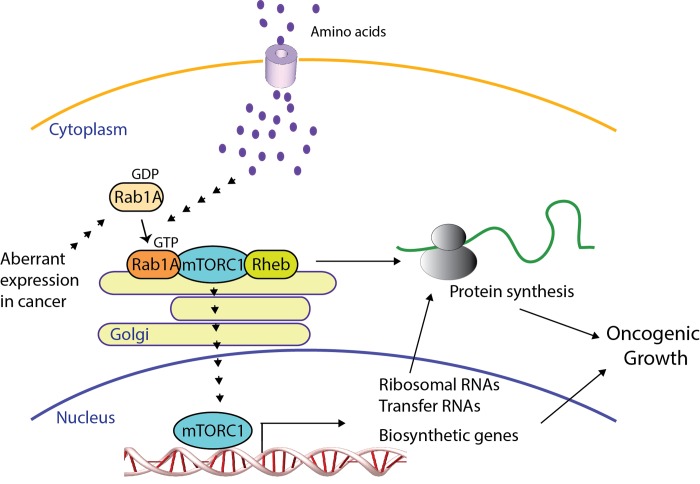
Role of RAB1 in amino acid-Mtorc1 signaling and oncogenic growth RAB1 mediates amino acid signaling to stimulate mTORC1 activity by recruiting Rheb to mTORC1 on the Golgi membranes, which results in elevated expression of transfer and ribosomal RNA and biosynthetic genes, as well as protein translation. RAB1 overexpression frequently occurs in human cancer, which enhances amino acid-mTORC1 signaling and oncogenic growth.

Rab1 is the founding member of the Rab small GTPase family, which is well known to mediate membrane trafficking between the endoplasmic reticulum (ER) and Golgi apparatus [1]. It is a highly conserved protein with two different isoforms, Rab1A and Rab1B in mammals, and predominantly localized on the membrane of endoplasmic reticulum (ER) and Golgi apparatus. The ER-Golgi membrane system is increasingly recognized for its role in anchoring cell signaling, where some key regulatory proteins such as mitogen receptors and transcription factors as are synthesized, modified and transported to the cell surface or nucleus. Consistently, some recent studies show that Rab1 is a regulator of several central signal transduction pathways, particularly mTOR pathway. A genetic screens in yeast identified Rab1 to be critical is for mediating amino acid (AA) signaling to activate mTORC1 [2]. The function of Rab1 in mTORC1 signaling was further investigated in yeast, human embryonic kidney (HEK) 293, and colorectal and liver cancer cells [2, 3]. AA was found to stimulate Rab1A interaction with mTORC1 in the Golgi in a GTP-dependent manner, which further promotes the binding of Rheb to and activation of mTORC1 (Figure 1). Golgi is known for transducing signals of nutrients such as cholesterol into the nucleus. That mTOR is also localized in the nucleus where it regulates gene expression [4] raises an interesting possibility that Rab1 is especially important for activating AA-mTOR signaling into the nucleus.

Consistent with Rab1's growth regulatory functions, RAB1 is commonly deregulated in human cancer. Overexpression of RAB1A and RAB1B is found in colorectal cancer (CRC) and hepatocellular carcinoma (HCC) [2, 3], as well as several other cancer types including tongue squamous carcinomas (TSCCs) [5]. RAB1A is overexpressed in 40 to 60% human colorectal and hepatocellular carcinomas, which is strongly associated with cancer progression and poor survival [2, 3]. Strikingly, the rate of overexpression in TSCCs is between 93 and 98% [5]. How RAB1A expression is deregulated has been investigated in HCC. In a study of 187 hepatitis B virus (HBV)-positive HCCs, increased gene copy number rather than changes in DNA methylation was found to be a contributor to RAB1A overexpression [3]. MicroRNAs (miRNAs) have also been shown to regulate RAB1 expression in human cancer. For example, miR-15b-5p is down-regulated, which results in elevated expression of its target gene RAB1A in HCC [6].

Studies with in vitro and in vivo tumor models demonstrated that RAB1A is a potent oncogene. Moderate RAB1A overexpression is sufficient to transform NIH3T3 cells [2]. Compared with the activated H-RAS^V12^ oncogene, RAB1A displays stronger transforming activity as measured by cell growth, colony formation and xenograft tumor assays. Rab1A overexpression specifically enhances mTORC1 signaling, tumor invasion and progression [2, 3]. Conversely, RAB1A knockdown selectively attenuates oncogenic growth and invasion of cancer cells with high RAB1A expression, while not significantly affecting those tumors with low RAB1A expression [2, 3]. Consistent with the role of Rab1A in AA signaling, RAB1A overexpression renders cancer cells more reliant on AA for their growth and survival, suggesting that these cancer cells have become addictive to AA-mTORC1 signaling pathway signaling [2, 3]. Indeed, cancer cells with high RAB1A expression are hypersensitive to rapamycin, a highly selective mTORC1 inhibitor [2, 3]. Rapamycin analogs term-sirolimus and everolimus are US Food and Drug Administration (FDA)-approved drugs for treatment of renal and breast cancers. However, the overall response rate for these drugs is relatively moderate [7]. One major limiting factor is the lack of reliable biomarkers to predict the therapeutic response [7]. Thus RAB1 overexpression is potentially of value in tumor staging and prognostic analysis, as well as in guiding mTOR targeted therapy.
